# An update on vessel preparation in lower limb arterial intervention

**DOI:** 10.1186/s42155-020-00175-6

**Published:** 2020-11-27

**Authors:** William Ormiston, Shelagh Dyer-Hartnett, Rukshan Fernando, Andrew Holden

**Affiliations:** Department of Interventional Radiology, Auckland City Hospital, Auckland, New Zealand

**Keywords:** Vessel preparation, Peripheral arterial disease, Intravascular lithotripsy, Atherectomy, Cutting balloon, Scoring balloon, specialty balloon, chocolate balloon, Serranator balloon, spur stent, drug-coated balloon

## Abstract

**Background:**

Plain balloon angioplasty has traditionally been used to treat lower limb arterial disease but can be limited by significant residual stenosis, vessel recoil, dissection, and by late restenosis. Appropriate vessel preparation may significantly improve short and long-term outcomes. We aim to give an overview of some of the devices currently available, or under investigation, for vessel preparation in the lower limb.

**Main text:**

Vessel preparation devices include those that remove plaque (atherectomy devices) and those that modify plaque.

The four groups of plaque removing atherectomy devices are defined by their plaque removal method: Directional, rotational orbital and excimer laser are categories of devices investigated for plaque modification.

Intravascular lithotripsy devices generate sonic pulsatile pressure waves that pass into the vessel wall cracking calcified plaques whilst sparing soft tissue. This enables dilatation of calcified lesions at low pressure by conventional balloons and enables full stent expansion.

Other balloon based vessel preparation devices were designed to modify plaque and produce more controlled, lower pressure luminal expansion without major dissections and potentially with less recoil than conventional angioplasty balloons. Scoring balloons have a helical nitinol element attached to the balloon that scores plaque facilitating uniform luminal enlargement. Further specialty balloons have been developed in recent years, including the Chocolate, Phoenix and Serranator balloons. Finally, the temporary Spur self-expanding retrievable nitinol stent has a series of radially aligned spurs that are driven into the vessel wall by post-dilatation, potentially improving drug delivery.

**Conclusion:**

Lesion specific vessel preparation aims to improve both short and long term outcomes through improved penetration of anti-proliferative drug, maximising luminal gain, reducing the need for stent placement and minimising intimal injury. Some forms of vessel preparation appear to improve short term outcomes; long-term outcomes remain uncertain. An overview of some of the multiple devices available for vessel preparation is presented.

## Background

Endovascular therapy for peripheral artery disease (PAD) in the lower limbs is a viable alternative to surgical management but is limited by residual stenosis, recoil, dissection, thrombosis and restenosis (Norgren et al., 2007). Recent advances in vessel preparation aim to improve short and long term outcomes by a number of mechanisms. These techniques involve debulking or modifying plaque, with the hope of reducing barotrauma from subsequent angioplasty and improving arterial wall penetration of anti-proliferative drugs from drug-eluting devices (Tzafriri et al., 2017; Babaev et al., 2015), the benefits of which are well established, particularly in the femoropoliteal segment (Rosenfield et al., 2015). Further potential benefits include the reducing the need for stent deployment by attempting to facilitate vessel expansion, especially in the heavily calcified vessel, aiding in stent expansion when required and treating any potential instent restenosis (Katsanos et al., 2017). Whilst no large randomized trial data are available, atherectomy has potential as a useful tool in debulking in the lower limb vasculature (Katsanos et al., 2017), and it’s use has been investigated in combination with drug-coated balloons, with the hope of aiding uptake of drug (Gandini et al., 2013; Kokkinidis et al., 2018). Intravascular lithotripsy delivers locoregional sound waves to fracture vascular calcification in an effort to modify plaque (Safety and Performance of & nbsp;Lithoplasty for Treatment of Calcified Peripheral Artery Lesions, 2020). This has a number of potential benefits, especially in dilating highly calcified resistant stenoses (Holden, 2019). There are myriad other balloon based vessel preparation devices, among them the Angiosculpt (Philips, Amsterdam, Netherlands) (Kronlage et al., 2020), Serranator (Cagent Vascular, Wayne, PA, USA) (Holden et al., 2019) and Chocolate Balloon (TriReme Medical, Pleasanton, CA, USA) (Shishehbor et al., 2016), which use diverse mechanisms to increase luminal gain and/or improve subsequent drug delivery. Many of these devices remain in the evaluation stage. The aim of this short review is to provide a brief overview of some of the new technologies available for vessel preparation, and their potential roles in management of PAD.

## Atherectomy

Peripheral atherectomy devices debulk atheroma in contrast to balloon angioplasty and stenting where the atheroma is displaced outwards and longitudinally (Katsanos et al., 2017). The goal of this therapy is to aid treatment of heavily calcified lesions and possibly improve drug delivery and reduce the need for stents, particularly in suboptimal locations for stent such as the popliteal and common femoral artery. Although the evidence is currently limited, atherectomy plus drug-coated balloon treatment has been compared with drug-coated balloon alone in patients with peripheral vascular disease, in both de novo (Kokkinidis et al., 2018; Zeller et al., 2017; Cioppa, 2018; Cioppa et al., 2017) and instent restenotic stenoses (Gandini et al., 2013; Sixt et al., 2013). This combination, if effective, could be particularly useful in areas where stent use should be minimized, such as the common femoral artery, popliteal artery, ostial lesions and heavily calcified lesions (Zeller et al., 2017). One must bear in mind, concerns have been raised in regards to higher complication rates in patients who received atherectomy (with or without PTA) in comparison to PTA alone, with one review finding a higher incidence of amputation and other major adverse events (Ramkumar et al., 2019). Consideration should also be given to the increased sheath sizes required for many of the devices and added cost over and above standard plain and drug coated balloons (Katsanos et al., 2017). Since the first rotational atherectomy devices were introduced in the 1980s (Höfling et al., 1987), multiple new designs have been developed, however there remains no data directly comparing the different available devices (A Critical View of the Peripheral Atherectomy Data in the Treatment of Infrainguinal Arterial Disease, 2012). These can be divided into four groups according to the method of plaque removal and include directional atherectomy (Cioppa, 2018), excimer laser atherectomy (Gandini et al., 2013; Kokkinidis et al., 2018), rotational atherectomy (Beschorner et al., 2013) and orbital atherectomy (Shammas et al., 2012a; Das et al., 2014; Safian et al., 2008).

**Fig. 1:**
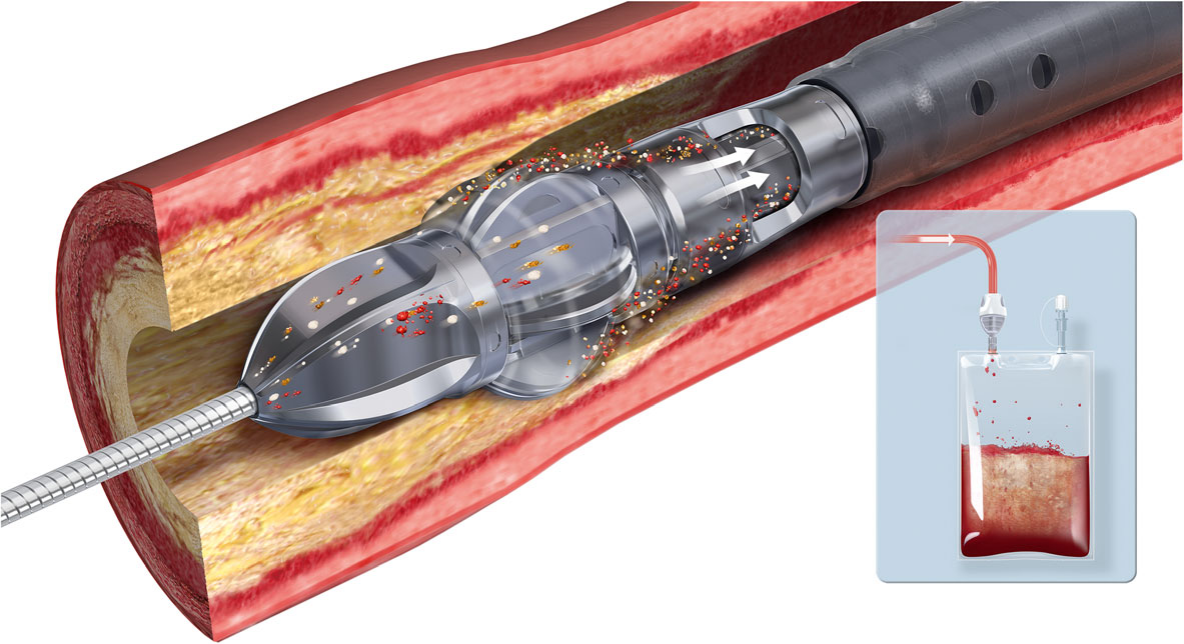
Jetstream Atherectomy Device: The Jetstream rotational, directional atherectomy device, demonstrating active debris collection via the aspiration port in the nose cone, and deposition into collection bag (inset). Reproduced with permission (Boston Scientific)

**Fig. 2:**
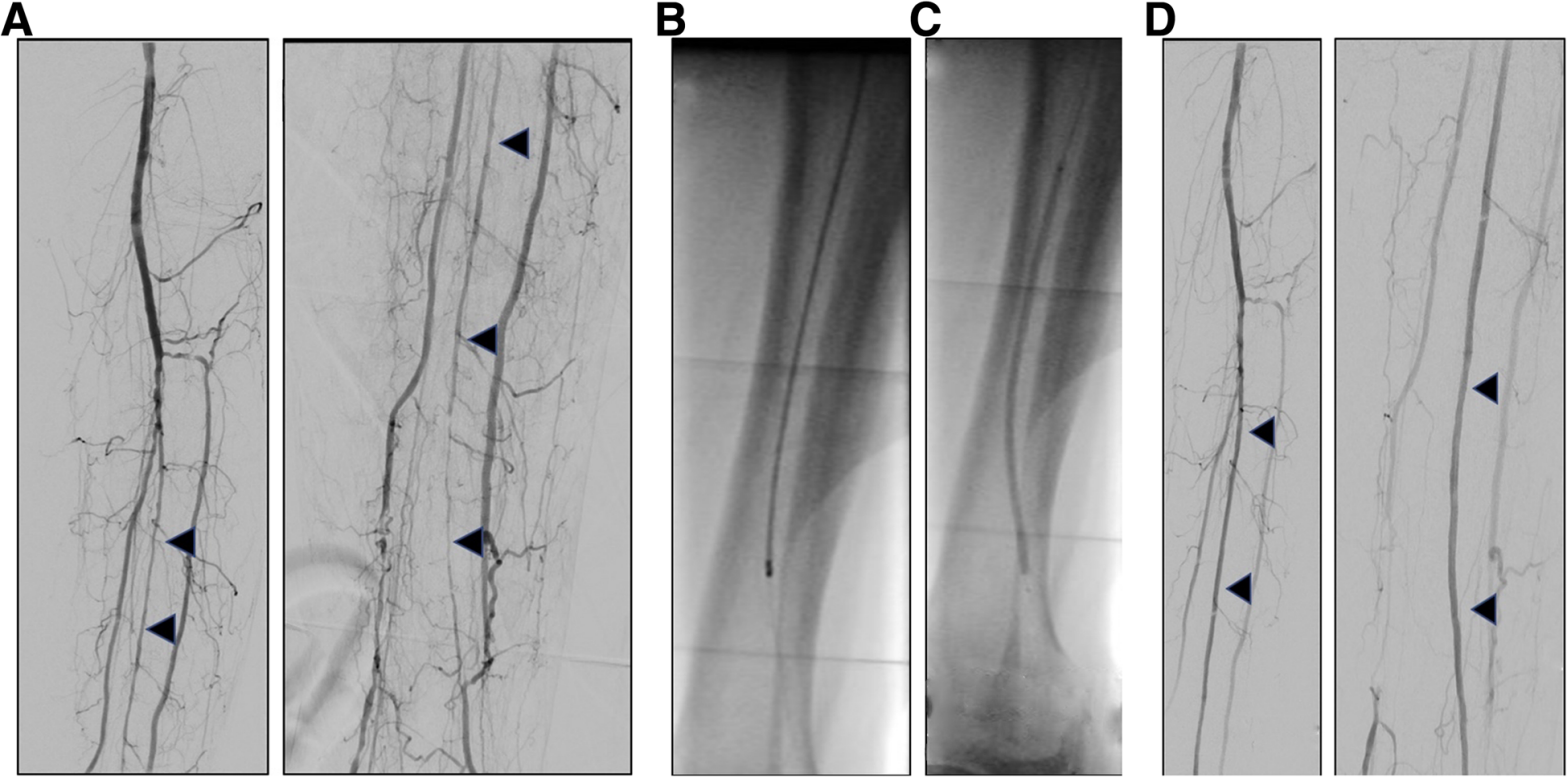
Clinical images pre, during and post atherectomy: Peroneal artery long segment atheroma (**a**), treated with Jetstream atherectomy (**b**) and DCB (**c**), resulting in patent vessel (**d**)

Directional atherectomy employs a side-cutting rotating blade to excise plaque which is collected in the nose cone. The catheter is directed alongside the plaque of interest and the device is activated, and is of particular use in eccentric plaque (Katsanos et al., 2017). The nose cone must be emptied after a few passes once the nose cone becomes full, making this technique potentially more time consuming that other similar atherectomy devices. Devices include the Hawk system, such as the SilverHawk, TurboHawk and HawkOne (Medtronic, MN, USA), see Fig. 1. The non-randomized DAART trial (directional atherectomy and anti-restenotic therapy vs drug-coated balloon alone in the popliteal) showed a trend towards increased 12 month primary patency (82% versus 65%, *p* = 0.021) for the DAART arm (Stavroulakis et al., 2017), however aneurysmal dilatation was seen more often after DAART (although this was not statistically significant). Furthermore, while the DEFINITIVE AR atherectomy pilot trial of directional atherectomy with either the SilverHawk or TurboHawk devices followed by paclitaxel coated balloon (Cotavance, Germany) therapy showed superior outcomes initially compared with a drug-coated balloon alone, the trial was not powered to and also did not show differences at 12 months (see Fig. 2).

Excimer laser atherectomy employs ultraviolet radiation to disintegrate atheroma from the arterial lumen without heating, and is commonly indicated for both de novo and, especially, instent restenosis (Katsanos et al., 2017). The catheter should be advanced slowly with saline flushing to remove energy absorbing blood and contrast from the vessel. In combination with drug coated balloons (DCB), laser atherectomy demonstrated a higher 1-year patency rate in two separate recent studies. One randomized trial where atherectomy plus DCB was compared with DCB alone in the management of instent restenosis/occlusion (66.7% vs 37.5%) (Gandini et al., 2013) and one retrospective study of atherectomy and DCB of de novo lesions in comparison with atherectomy in combination with plain balloon angioplasty (86.7% vs 56.9%) (Kokkinidis et al., 2018).

In rotational atherectomy plaque is ground by a high speed concentrically rotating tip (burr). The Peripheral Rotablator (Boston Scientific, MN, USA) has a burr coated distally with diamond chips, that macerate atheroma into debris smaller than red blood cells which embolizes. The Pathway Jetstream (Boston Scientific) is a over-the-wire cutting rotational atherectomy system, which fits through a 7 Fr sheath, and has the ability to actively aspirate during use, thereby reducing procedure time **(**Fig. 1**)**. Luminal diameter gain is limited by burr diameter so if a larger lumen is desired, the burr needs to be swapped out for a larger one. Other rotational devices include the Phoenix device (AtheroMed, CA, USA). An European multicentre prospective registry (Beschorner et al., 2013) of the Pathway system demonstrated improved ankle brachial index and Rutherford scores yet low primary patency rates of 33 and 25% at 12- and 24-month follow-up.

The Diamondback 360° (Cardiovascular Systems Inc., MN, USA) is the only commercially available orbital atherectomy device, which employs the orbital rotation of a diamond coated crown to macerate plaque; a technique that is similar to rotational atherectomy. The device therefore enables circumferential plaque removal, and the volume of debulking increases with increased rotational speed. There is no aspiration and distal embolization is possible. Evidence is accumulating that orbital atherectomy is also useful in calcified disease, particularly in the infrapopliteal segment (Shammas et al., 2012a; Das et al., 2014; Safian et al., 2008).

The Pantheris device (Avinger Inc., CA, USA) is an over-the-wre catheter which with a combination of optical coherence tomography (OCT) and a more traditional directional atherectomy device, with the goal of aiding the operator in targeting the appropriate plaque to be treated, whilst minimising damage to the non-diseased wall. The nose cone must also be emptied on a regular basis for this device, similar to the other directional atherectomy devices. Safety and efficacy has been recently demonstrated, with a high primary efficacy (97% of the 198 lesions treated), with no significant perforations and 1 catheter rated dissection (0.5%) (Schwindt et al., 2017).

All atherectomy devices probably carry some risk of distal embolization of plaque debris, therefore distal filter protection should be considered. The Emboshield NAV6 (Abbott Vascular, CA, USA) is usually recommended for rotational atherectomy devices such as the Jetstream, and SpiderFX (ev3, MN, USA) usually recommended with directional devices (Shammas et al., 2012b). Vasodilators should also be considered to counteract vasopasm in response to plaque modification (Franzone et al., 2012).

## Intravascular lithotripsy (IVL)

**Fig. 3:**
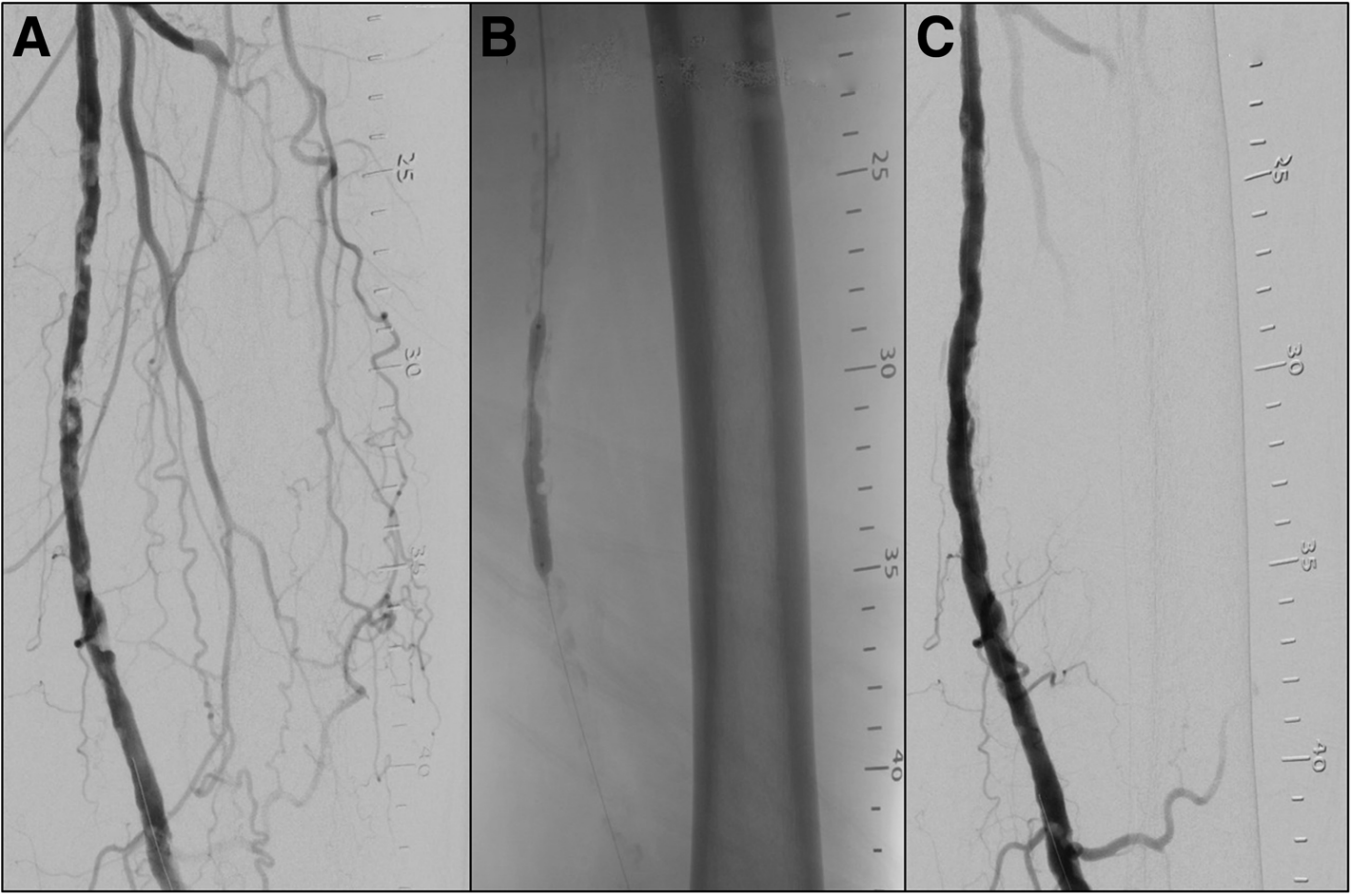
Intravascular Lithotripsy: Angiography demonstrating heavy calcific disease in the SFA (**a**). Partial inflation of the IVL device through the stenotic disease (**b**). Post intravascular lithoplasty with a 5.5 mm balloon catheter, resulting in a significantly improved luminal diameter with non-significant dissection (**c**)

The Shockwave (Shockwave Medical, CA, USA) intravascular lithotripsy device (IVL), utilises an over-the-wire angioplasty balloon, optimally sized at 1.1:1, containing emitters, which generate electro-hydraulic sonic waves (Fig. 3). This balloon is inflated to 4 atm, allowing apposition to the calcification. The sonic waves are then generated, causing micro-fractures within the intimal and medial wall calcification, whilst sparing the soft tissue (Holden, 2019), and this process is repeated in an overlapping fashion. This results in improved vessel compliance allowing luminal gain with reduced force (Brodmann et al., 2017). The technology also disrupts the calcific barrier and potentially improves anti-proliferative drug delivery and thereby its effect, which is currently under investigation.

In the Disrupt PAD I & II small single arm trials, IVL achieved initial results with 78% resulting in a < 30% residual stenosis, and only one patient requiring stenting for dissection in the combined cohort of 95 patients (Brodmann et al., 2019). During Disrupt PAD I & II, the use of drug-eluting technology was not allowed in conjunction with IVL, and it is likely that this combination would significantly improve long-term results, considering the known benefits of DCBs (Rosenfield et al., 2015). Therefore, the DISRUPT PAD III trial, which has recently completed recruitment, will answer this question, as it is a randomized controlled trial of 400 patients with moderate/severely calcified femoro-popliteal arteries treated with either IVL plus drug-coated balloon or plain balloon plus drug-coated balloon. In addition to utilizing IVL as an adjunct before DCB, it may prove significantly useful in heavily calcified lesions as a vessel preparation tool for implantation of a Supera stent, which requires further study.

## Other balloon-based devices

Balloon based vessel preparation devices use different mechanisms to improve initial outcomes compared with plain balloon angioplasty.

The AngioSculpt device (Philips, Amsterdam, Netherlands), consists of a non-compliant balloon with an external helical nitinol element that scores plaque, avoiding slippage and offering controlled, predictable dilatation. The PANTHER single centre registry trial of 124 patients suggested value of this device for short heavily calcified SFA lesions (Lugenbiel et al., 2018). Other balloon-based devices such as the Flextome Cutting Balloon (Boston Scientific) and Ultrascore (Bard Medical Division, Covington, GA) lower balloon dilatation pressure needed to expand stenoses by scoring, cracking and fracturing calcified plaque using microblades or wires set on the balloon.

**Fig. 4:**
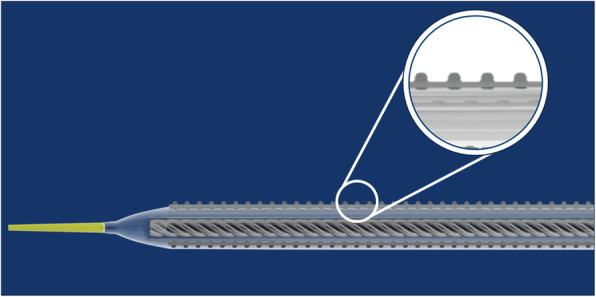
Serranator balloon: The Serranator balloon (Cagent Vascular, Wayne, PA, USA) is a semicompliant balloon with four serrated embedded strips along the longitudinal axis (see inset) which penetrate the intima which may improve delivery of drug to the vessel wall

The serrated strips embedded in the Serranator balloon (Cagent Vascular, Wayne, PA) create a controlled, uneven distribution of pressure (Fig. 4). These serrations crack the plaque and penetrate into the intima and possibly media, achieving greater vessel compliance at lower pressures even in complex calcified lesions, leading to improved long-term patency (Tzafriri et al., 2017). The serrations may also improve anti-proliferative drug penetration into deeper vessel wall layers and therefore promote greater drug efficacy. The safety and efficacy of the Serranator has been evaluated in the femoro-popliteal circulation in the small prospective, multicentre, single-arm PRELUDE study, with no patients experiencing adverse events (Holden et al., 2019). Primary patency was 64% at 6 months, with the 6 month Rutherford category showing significant improvement.

**Fig. 5:**
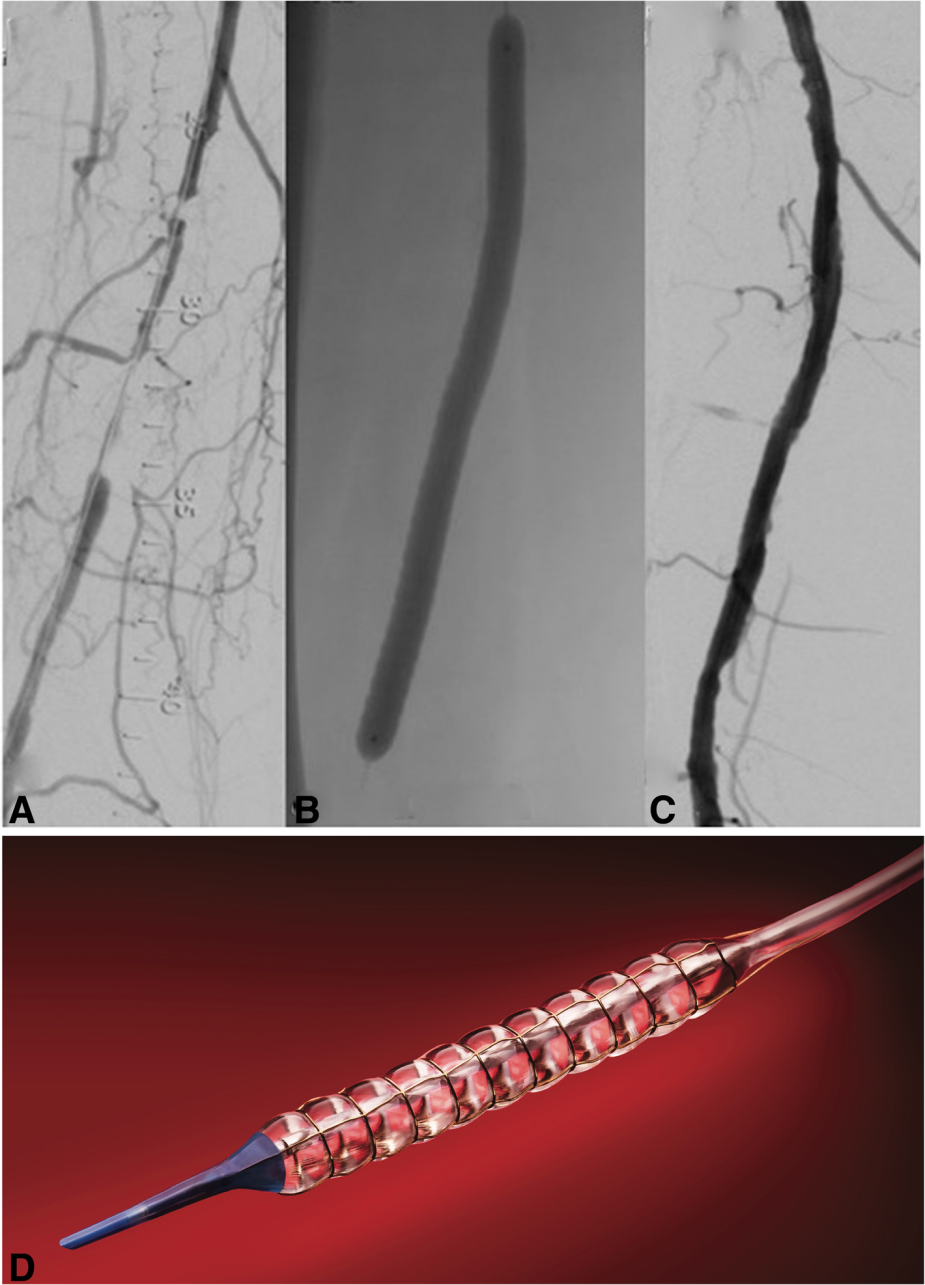
Chocolate DCB: Treatment of a chronic superficial femoral artery occlusion (**a**) using the Chocolate DCB. A magnified image of the balloon over the target lesion (**b**) allows visualisation of the “pillow and grooves” effect. Subtracted angiogram showing the result after primary angioplasty (**c**). Diagrammatic representation of the nitinol cage surrounding the balloon with resulting “pillows and grooves” (**d**, reproduced with permission, Medtronic)

The special features of the Chocolate Balloon (TriReme Medical LLC, CA, USA) are designed to decrease dissection rates (Figs. 5, 31]. The balloon has a nitinol constraining cage that expands with balloon expansion and shields the vessel wall from the torsional stress of balloon unwrapping during inflation. It also creates “pillows” and “grooves” that provide stress relief points that allow for plaque modification, limit dissection and at the same time increase the contact surface area to 120%. The Chocolate PTA balloon catheter demonstrated excellent procedural outcomes with low dissection rates and bailout stent use in a prospective registry (Mustapha et al., 2018), and the antiproliferative drug-coated version (Chocolate Touch) had almost 90% 12 month patency in the small single arm ENDURE trial of complex lesions (Shishehbor et al., 2016). The VascuTrak device (BARD, NJ, USA) is a similar scoring balloon, characterised by two longitudinal wires along the length of the balloon causing partial constraint, which has been evaluated in a safety and efficacy trial with good success (Baumhäkel et al., 2018).

The Temporary Spur Stent System (Reflow Medical, San Clemente, CA), a self-expanding, retrievable nitinol stent with a series of radially aligned spurs that are driven into the vessel wall by post-dilatation. This system potentially improves drug delivery, and is still in trial stages (DEEPER OUS). A retrievable self-expanding stent with balloon-expanding spikes creates channels deep in the tibial vessel wall, possibly allowing for better drug penetration into the media and adventitia.

## Conclusions

There are multiple new technologies available for vessel preparation in the lower limb, for example atherectomy, intravascular lithotripsy and balloon-based vessel preparation devices. Some forms of vessel preparation appear to improve short term outcomes; long-term outcomes remain uncertain. Further evidence is awaited, with particular interest in regards to randomised comparisons between plain angioplasty and these new technologies with and without adjunctive anti-proliferative drug-coated balloons in adequately powered trials.

## Data Availability

Not applicable

## Abbreviations

PAD: Peripheral Artery Disease
IVL: Intravascular Lithotripsy
